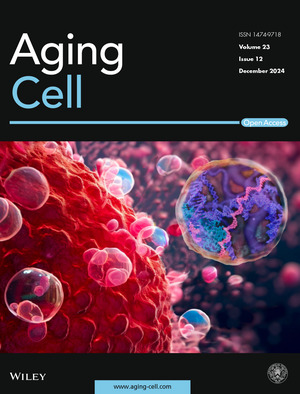# Featured Cover

**DOI:** 10.1111/acel.14454

**Published:** 2024-12-11

**Authors:** Kavita Singh, Shraddha I. Khairnar, Akshay Sanghavi, Tanuja T. Yadav, Neha Gupta, Jay Arora, Harold L. Katcher

## Abstract

Cover legend: The cover image is based on the article *E5 treatment showing improved health‐span and lifespan in old Sprague Dawley rats* by Kavita Singh et al., https://doi.org/10.1111/acel.14335.